# Assessment of Esophagogastric Junction Barrier Function With the Supine‐Upright Transition of the Chicago Classification Protocol

**DOI:** 10.1111/nmo.70088

**Published:** 2025-05-26

**Authors:** Stefano Siboni, Roberta De Maron, Andrea Pasta, Marco Sozzi, Francesco Calabrese, Pierfrancesco Visaggi, Nicola De Bortoli, Anthony Hobson, Jordan Haworth, Daniele Bernardi, Takahiro Masuda, Giovanni Aldinio, Marina Coletta, Roberto Penagini, Edoardo Savarino, Emanuele Asti, C. Prakash Gyawali, Elisa Marabotto

**Affiliations:** ^1^ Division of General and Emergency Surgery, IRCCS Policlinico San Donato, Milano University of Milan Milan Italy; ^2^ Gastroenterology Unit IRCCS Policlinico San Martino Genoa Italy; ^3^ Department of Internal Medicine University of Genoa Genoa Italy; ^4^ Division of Gastroenterology, Department of Traslational Research and New Technologies University of Pisa Pisa Italy; ^5^ The Functional Gut Clinic London UK; ^6^ Department of Surgery Jikei University School of Medicine Tokyo Japan; ^7^ Department of Pathophysiology and Organ Transplantation University of Milan Milan Italy; ^8^ Gastroenterology and Endoscopy Unit Fondazione IRCCS Ca' Granda Ospedale Maggiore Policlinico Milan Italy; ^9^ University of Milan Milan Italy; ^10^ Division of Gastroenterology, Department of Surgical, Oncological and Gastroenterological Sciences University of Padua Padua Italy; ^11^ Division of Gastroenterology Washington University School of Medicine St. Louis Missouri USA

**Keywords:** gastro‐esophageal reflux disease, high‐resolution manometry, intra‐abdominal pressure, straight‐leg raise maneuver

## Abstract

**Background & Aims:**

The straight leg raise (SLR) is a provocative maneuver used to assess the esophagogastric junction (EGJ) barrier function during high‐resolution manometry (HRM) and is part of the Milan Score (MS). The Chicago Classification 4.0 (CCv4.0) protocol requires patients to perform a supine‐upright transition (SUT), increasing intra‐abdominal pressure (IAP). The aim of this study was to compare the SUT and SLR maneuvers for efficacy in increasing IAP and in predicting pathologic gastroesophageal reflux disease (GERD).

**Methods:**

Consecutive adult patients with persistent GERD symptoms undergoing HRM and pH‐impedance were prospectively enrolled. After completion of the supine swallows of the CCv4.0 protocol, the SLR maneuver was performed and the patients were asked to get up to the upright position (SUT). IAP and intra‐esophageal pressure (IEP) were recorded at baseline and during the maneuvers. GERD was defined as acid exposure time > 6% according to Lyon 2.0.

**Results:**

Among the 110 patients included (age 55 years; 59.1% female, BMI 25.4 kg/m^2^) SUT was effective in 94 and SLR in 85. SUT was more sensitive than SLR (77.4% vs. 71.0%) but less specific (63.5% vs. 79.6%) in predicting GERD. On ROC analysis, the AUC of the MS‐SUT was 0.825 and MS‐SLR 0.854. When both maneuvers were effective (73 patients) SUT predicted GERD in 69.9%, SLR in 76.7% (*p* = 0.192). When concordant (52 patients) sensitivity and specificity were 88% and 80%, and the AUC of the MS was 0.872.

**Conclusions:**

SUT is comparable to SLR, with higher sensitivity but lower specificity. When both are concordant, the SUT can strengthen confidence in SLR and increase the accuracy of the Milan Score.

## Graphical abstract

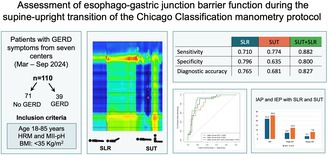

**Summary**

- The SUT maneuver was comparable to the SLR in terms of IAP increase and GERD prediction. When both maneuvers were effective and concordant, they demonstrated excellent diagnostic accuracy.
- The SUT maneuver could potentially simplify the HRM protocol by utilizing a position change that is already required, thereby reducing examination time and patient discomfort.

## 1 Introduction

High‐resolution manometry (HRM) plays a crucial role in the diagnostic work‐up of patients with reflux symptoms by enabling precise pH‐catheter positioning and excluding conditions that mimic gastro‐esophageal reflux disease (GERD) symptoms, such as esophageal achalasia [1]. Beyond these basic roles, HRM provides comprehensive assessment of esophagogastric junction (EGJ) morphology and anti‐reflux barrier disruption [2]. Hiatal hernia, one of the main predisposing factors of pathologic acid exposure time (AET), can be categorized using various classification systems with varying relationships to AET [3, 4, 5, 6, 7, 8, 9]. Ineffective esophageal motility (IEM) impacts esophageal clearance of refluxate and thereby correlates with GERD [10, 11, 12]. Further, both anatomic and functional characteristics of the lower esophageal sphincter (LES), including pressure and length, have consistently been demonstrated to participate in GERD pathogenesis [13, 14, 15].

In recent decades, novel parameters and provocative maneuvers have been introduced to enhance the diagnostic potential of HRM in GERD evaluation. The EGJ‐contractile integral (EGJ‐CI) provides improved assessment of EGJ barrier function [16]. Provocative maneuvers include multiple rapid swallows (MRS) which measure peristaltic reserve [17], and the straight leg raise (SLR) maneuver, which enables dynamic evaluation of EGJ response to increased intra‐abdominal pressure (IAP) [18, 19, 20, 21].

Abnormal response to the SLR maneuver associates with abnormal reflux burden, and this has been successfully utilized in clinical and research protocols using HRM to predict pathologic GERD [18]. A recent multicenter study established normative SLR metrics and further validated its predictive capability for GERD [19]. The SLR is the primary component of the Milan Score, a novel HRM metric that integrates four GERD‐related parameters to quantify anti‐reflux barrier disruption [22]. However, SLR is an additional provocative maneuver that adds to the complexity of the modern HRM protocol, and an alternate, efficient method of IAP increase could be of value.

The Chicago Classification 4.0 (CCv4.0) protocol includes a transition from supine to upright position, and this naturally increases IAP through engagement of core muscles during positional change, potentially mimicking the SLR maneuver, which elevates IAP via active abdominal muscle contraction. Both the SLR and the supine‐upright transition (SUT) maneuvers may challenge the EGJ by creating a “common cavity” effect, where increased intra‐gastric pressure promotes reflux in the presence of a disrupted EGJ barrier [20]. While the SLR has been validated for GERD prediction [19], no prior studies have evaluated SUT as a diagnostic maneuver. We hypothesize that IAP increase and EGJ response during the SUT could mimic or complement the SLR response. The aim of this study was to compare the SUT and SLR maneuvers for efficacy in increasing IAP and in predicting pathologic GERD.

## 2 Methods

### 2.1 Study Design and Population

A prospective multicenter study involving seven high‐volume tertiary care centers across Europe, North America, and Asia that enrolled consecutive patients between March and September 2024 was performed to compare SUT and SLR maneuvers. Inclusion criteria consisted of age between 18 and 85 years, HRM and multichannel intraluminal impedance‐pH (MII‐pH) performed off proton pump inhibitors (PPI) within 2 weeks of each other for persistent GERD symptoms, and the ability to perform the SLR and SUT maneuvers as part of the HRM protocol. Patients with previous foregut surgery, severe obesity (body mass index (BMI) > 35 kg/m^2^), para‐esophageal hiatal hernia, scleroderma, eosinophilic esophagitis, and major motility disorders were excluded. Patients with BMI > 35 kg/m^2^ were excluded to minimize confounding effects of excessive IAP, which could disproportionately affect EGJ function and bias the diagnostic performance of SUT and SLR in a more representative GERD population. Patients with paraoesophageal hiatal hernias were excluded due to their complex anatomy, which may confound EGJ function assessment. Type I (sliding) hiatal hernias, where the EGJ and proximal stomach herniate into the thorax, were included, as they are common in GERD and relevant to this study. The study protocol was approved by the internal review board at each participating center and was conducted following the principles of the declaration of Helsinki.

### 2.2 Esophageal High‐Resolution Manometry

High‐resolution manometry was performed by experienced physicians or nurses after an overnight fast, using a solid‐state catheter assembly with circumferential pressure transducers spaced at 1‐cm intervals and each institution's preferred HRM system and following the standard CCv4.0 protocol [3]. To assess esophageal body peristalsis, ten single water swallows (5 mL) were performed in the primary position (supine or semi‐recumbent), followed by five swallows in the secondary position. The pressure topography of swallows was evaluated according to the current CCv4.0 criteria, sorting each swallow into intact, weak, or failed based on the distal contractile integral (DCI) (> 450, 100–450, and < 100 mmHg*cm*sec, respectively).

IEM was defined as > 70% weak swallows or ≥ 50% failed swallows. LES characteristics recorded consisted of total and intra‐abdominal length, basal pressure, EGJ‐CI, and EGJ morphology. EGJ‐CI was calculated during the reference period using the DCI toolbox across the EGJ, and corrected for respiratory cycle duration [16]. EGJ morphology was defined considering the LES–crura diaphragm (CD) separation, with three subcategories: type 1: superimposed LES and CD; type 2: LES‐CD separation < 3 cm; type 3 LES‐CD separation ≥ 3 cm. For the purpose of this study, other HRM provocative maneuvers such as multiple rapid swallows and rapid drink challenge were not included in the analysis.

### 2.3 Straight Leg Raise and Supine‐Upright Transition Maneuvers

The SLR maneuver was performed in the supine position as previously described [19], with double leg raise utilized when augmentation of IAP was unsatisfactory with the raising of just one leg. Following the SLR maneuver, patients were asked to return to the upright position without assistance from the operator and without using their hands (SUT maneuver). Both maneuvers were excluded and repeated if the patient demonstrated accidental swallowing during the maneuvers.

Mean IAP was recorded 1 cm below the CD and IEP 5 cm above the LES at baseline as well as during SLR and SUT. Both maneuvers were considered effective if the IAP increased by 50% compared to baseline. An increase of the peak IAP of at least 11 mmHg was considered pathologic, as per our previous report [19].

The Milan Score was computed using the official online calculator (www.milanscore.com), which integrates four key parameters: EGJ morphology, EGJ‐CI, IEM, and SLR maneuver response. For this study, we also computed the Milan Score using the SUT response, using the same online calculator. A Milan Score > 137 (risk rate 50%) was considered abnormal [22]. The diagnostic accuracy of the Milan Score was then calculated independently from the SUT and SLR maneuvers.

### 2.4 Esophageal pH‐Impedance Study

Ambulatory 24‐h pH impedance monitoring was performed off‐therapy after positioning the distal pH sensor of the probe 5 cm above the manometrically identified LES.

Tracings were reviewed after excluding meal periods, and the following data were recorded: total, upright, and recumbent AET, number of acid, weakly acid, and weakly alkaline reflux episodes, and DeMeester score [23]. Data were analyzed using dedicated software using Wingate consensus criteria [24]. Mean nocturnal baseline impedance (MNBI) was also acquired from the pH‐impedance studies [25].

Objective GERD was defined as biopsy proven long segment Barrett's esophagus or Los Angeles grades B, C, or D esophagitis at endoscopy, or AET > 6% on pH‐impedance monitoring [26]. Inconclusive GERD (AET 4%–6%) was designated pathologic if Lyon 2.0 adjunctive criteria were present (total number of reflux episodes > 80/day, MNBI < 1500 Ω, or reflux‐symptom association). GERD was excluded when AET < 4% [27].

### 2.5 Statistical Analysis

Continuous variables are reported as median and interquartile range (IQR), while categorical variables are reported as numbers and percentages. The normality of continuous variables was assessed using the Shapiro–Wilk test. The nonparametric Kruskal–Wallis test was used to compare continuous variables, while categorical variables were compared using the Chi Square test or Fisher test as appropriate. GERD and no‐GERD groups were compared in terms of demographics, HRM, and pH‐impedance variables. Median IAP increase and the performance of the SUT and SLR maneuvers in predicting pathologic GERD were calculated and compared. The diagnostic accuracy of the Milan Score was calculated independently with SUT and SLR using receiver operating characteristics (ROC) analysis. A McNemar test was performed to compare the diagnostic performances of the two maneuvers. A sub‐analysis of the subset of patients with effective both SUT and SLR maneuvers was performed. A two‐tailed *p*‐value < 0.05 was considered significant for all statistical tests. Statistical analyses were performed using R studio version 4.4.2.

## 3 Results

Among the 110 included patients (median age 55 years, 59.1% female, median BMI 25.4 kg/m^2^), 39 (35.5%) had conclusive GERD according to Lyon 2.0. Patients in the GERD group had a significantly higher BMI (28.0 vs. 23.9, *p* < 0.001), presented more frequently with typical symptoms (71.8% vs. 49.3%, *p* = 0.027), were more often on PPI (89.7% vs. 73.2%, *p* = 0.033), had a higher rate of hiatal hernia (63.9% vs. 36.4%, *p* = 0.012) and manifested erosive esophagitis more often (35.3% vs. 10.9%, *p* = 0.006) compared to patients in the no‐GERD group (Table 1).

**TABLE 1:** Demographic and endoscopic characteristics of the study population. Continuous values are expressed as median [IQR].

	Total (*n* = 110)	No GERD (*n* = 71)	GERD (*n* = 39)	*p*
Male, *n* (%)	45 (40.9)	31 (43.7)	14 (35.9)	0.544
Age (years)	55 [22]	55 [21]	55 [20]	0.098
BMI (kg/m^2^)	25.4 [6.3]	23.9 [5.5]	28.0 [5.9]	< 0.001
Symptoms duration (months)	36 [63]	36 [44]	36 [87]	0.416
Primary typical symptoms, *n* (%)	63 (57.3)	35 (49.3)	28 (71.8)	0.027
Primary extra‐esophageal symptoms, *n* (%)	47 (42.7)	36 (50.7)	11 (28.2)	0.027
PPI use, *n* (%)	87 (79.1)	52 (73.2)	35 (89.7)	0.033
Response to PPI				0.408
no response, *n* (%)	31 (34.8)	22 (40.7)	9 (25.7)	
partial response, *n* (%)	44 (49.4)	24 (44.4)	20 (57.1)	
full benefit, *n* (%)	14 (15.7)	8 (14.8)	6 (17.1)	
Endoscopic findings				
Hiatal hernia, *n* (%)	47 (46.1)	24 (36.4)	23 (63.9)	0.012
Esophagitis, *n* (%)	19 (19.4)	7 (10.9)	12 (35.3)	0.006
Questionnaires				
Gerd‐Q A	6 [6]	6 [6]	8 [5]	0.095
Gerd‐Q B	1 [4]	1 [4]	2 [3]	0.618
GERD‐HRQL	10 [18]	8 [17]	15 [20]	0.044
RSI	5.5 [15.0]	6 [15]	5 [13]	0.943

Abbreviations: BMI, Body mass index; GERD, Gastro‐esophageal reflux disease; GERD‐HRQL, GERD‐health related quality of life; PPI, Proton pump inhibitors; RSI, Reflux symptom index.

HRM and ambulatory reflux monitoring characteristics are summarized in Table 2. A hiatal hernia was found on HRM in 42% of the patients (28 patients with EGJ type 2 and 16 with EGJ type 3), with higher prevalence in the GERD group compared to the no‐GERD group (66.7% vs. 28.1%, *p* < 0.001). As expected, patients with GERD had a lower median EGJ‐CI (23.2 vs. 33.5 mmHg*cm, *p* = 0.046) and a higher AET (10.4% vs. 1.5%, *p* < 0.001).

**TABLE 2:** HRM and pH‐impedance characteristics of the study population. Continuous values are expressed as median [IQR].

HRM and pH data	Total (*n* = 110)	No GERD (*n* = 71)	GERD (*n* = 39)	*p*
HRM findings				
EGJ type				< 0.001
1, *n* (%)	66 (60.0)	52 (73.2)	14 (35.9)	
2, *n* (%)	28 (25.5)	17 (23.9)	11 (28.2)	
3, *n* (%)	16 (14.5)	2 (2.8)	14 (35.9)	
Hiatal hernia, *n* (%)	42 (42.0)	18 (28.1)	24 (66.7)	< 0.001
Hiatal hernia size (cm)	1 [1.8]	0 [1]	1.7 [1.5]	< 0.001
EGJ‐CI (mmHg × cm)	29.7 [43.6]	33.5 [44.9]	23.2 [45.1]	0.046
Patients with IEM, *n* (%)	31 (28.4)	15 (21.4)	16 (41.0)	0.045
pH‐impedance findings				
Acid exposure time (%)	3.3 [6.2]	1.5 [2.4]	10.4 [11.2]	< 0.001
Total reflux episodes, *n*	36 [36]	28 [35]	50 [33]	< 0.001
MNBI	2547 [2247]	3015 [1850]	1080 [1062]	< 0.001

Abbreviations: EGJ, Esophago‐gastric junction; EGJ‐CI, EGJ contractile integral; GERD, Gastro‐esophageal reflux disease; HRM, High‐resolution manometry; IEM, Ineffective esophageal motility; MNBI, Mean nocturnal baseline impedance.

**FIGURE 1:**
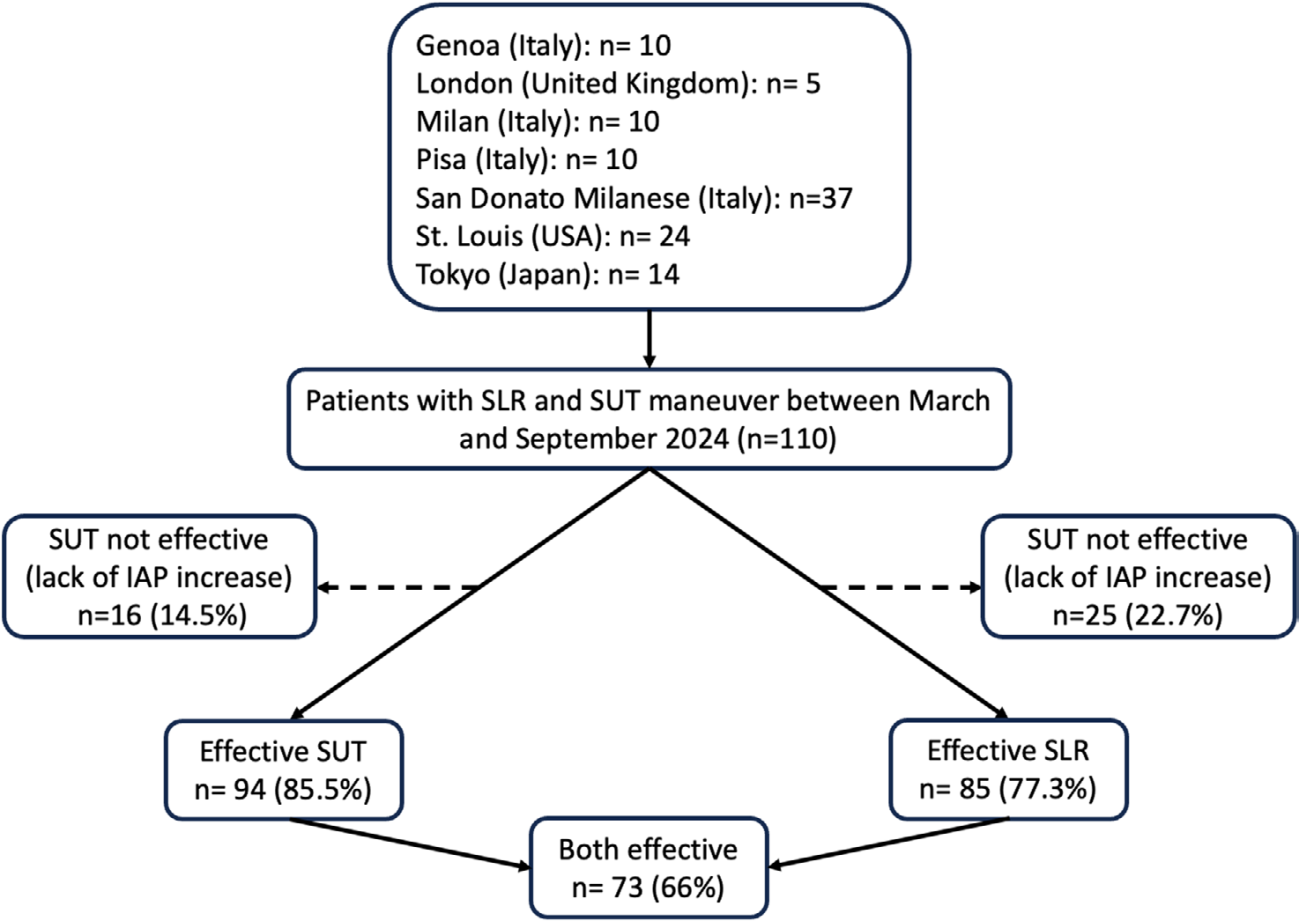
Flow chart of the study.

The flow chart of the study is shown in Figure 1. The SUT maneuver was effective (IAP increased by 50% compared to baseline) in 94 patients (85.5%), while the SLR was effective in 85 (77.3%). Demographic, clinical, and HRM characteristics between patients with effective and ineffective SUT were similar, while patients with ineffective SLR had a lower rate of hiatal hernia (24% vs. 48%, *p* = 0.045) compared with those with effective SLR (Figure S1).

**FIGURE 2:**
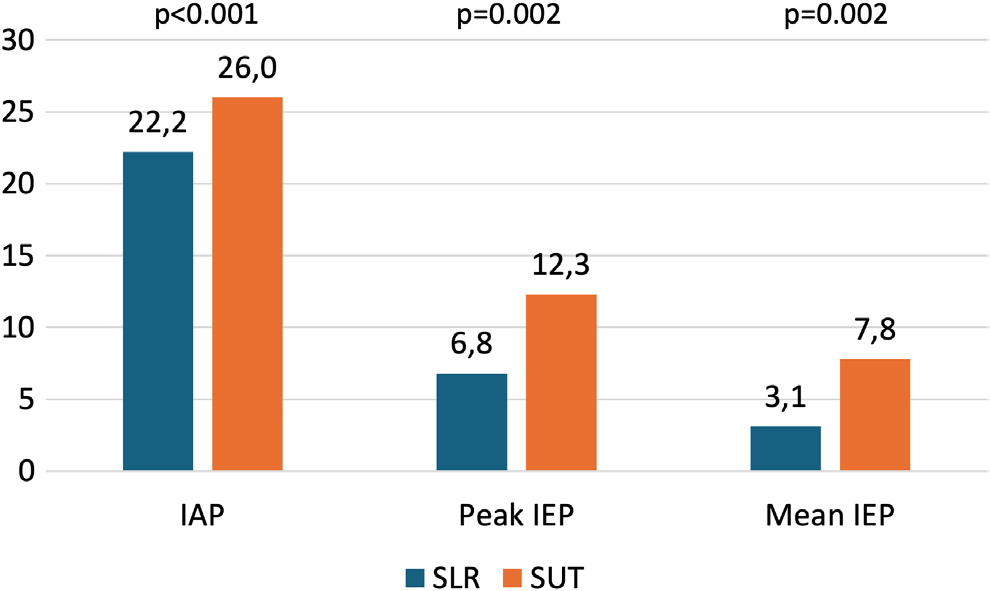
Intra‐abdominal pressure (IAP), peak and mean intra‐esophageal pressure (IEP) increase with the straight leg raise (SLR) and the supine‐upright transition (SUT).

**FIGURE 3:**
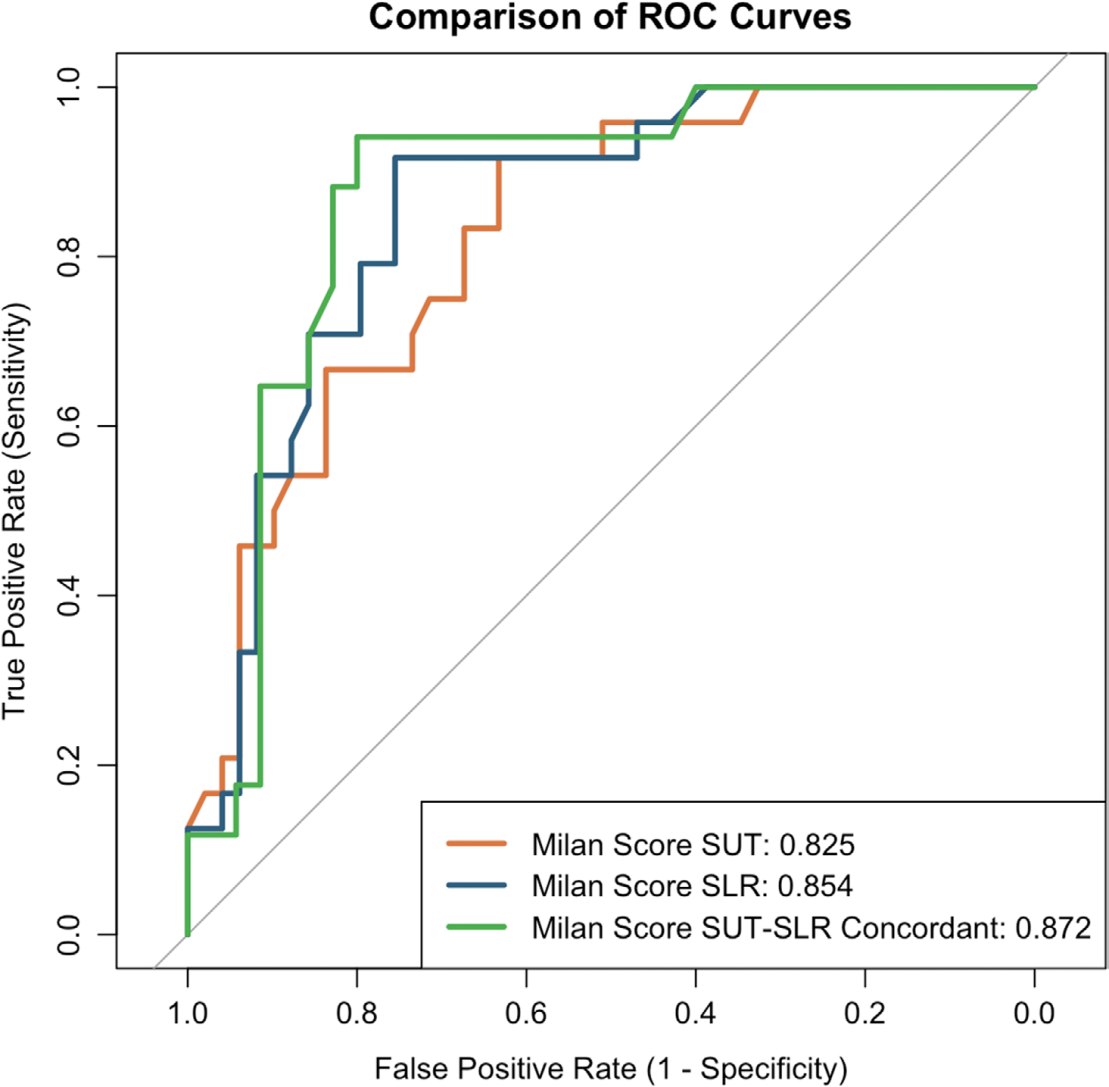
ROC analysis of the Milan Score calculated with SUT, SLR, and when SUT and SLR were concordant.

The SUT showed a greater increase in IAP, peak IEP, and mean IEP over the SLR (Figure 2). IAP increase was not influenced by BMI or age, as shown in Figure S2. Table 3 shows the performance metrics of the SLR and SUT maneuver, as measures of EGJ barrier function. The SUT was more sensitive than SLR (77.4% vs. 71.0%) but less specific (63.5% vs. 79.6%) in predicting pathologic GERD on pH‐impedance monitoring. However, diagnostic accuracy was higher with the SLR maneuver (76.5% vs. 68.1%). The overall diagnostic performance of the SUT and the SLR was comparable (*p* = 0.507). When the maneuvers were integrated into the Milan Score and the performance in predicting GERD was assessed through ROC curves, the Milan Score‐SUT had an area under the curve (AUC) of 0.825 (95% CI: 0.729–0.920) while Milan Score‐SLR had 0.854 (95% CI: 0.766–0.942) (Figure 3).

**TABLE 3:** Diagnostic performance of the SLR and the SUT. Variables are expressed as values (95% confidence interval).

	SLR	SUT
Sensitivity	0.710 (0.550–0.869)	0.774 (0.627–0.921)
Specificity	0.796 (0.689–0.904)	0.635 (0.516–0.754)
Positive predictive value	0.667 (0.506–0.828)	0.511 (0.368–0.654)
Negative predictive value	0.827 (0.724–0.930)	0.851 (0.749–0.953)
Diagnostic accuracy	0.765 (0.675–0.855)	0.681 (0.587–0.775)

Abbreviations: SLR, Straight leg raise; SUT, Supine‐upright transition.

Both maneuvers were effective in 73 (66%) patients. In this subgroup of patients, a positive SUT correctly identified pathologic GERD in 69.9% while SLR identified 76.7% (*p* = 0.192). When the two maneuvers were concordant (52 patients, 47%), sensitivity and specificity in predicting pathologic GERD were 88.2% and 80.0%, and the AUC of the Milan Score was 0.872 (95% CI: 0.771–0.974) (Figure 3).

## 4 Discussion

This study demonstrates that the SUT maneuver, which is mandatory during the CCv4.0 protocol, can mimic the SLR maneuver in assessing EGJ barrier function, which may shorten the HRM study protocol without loss of useful information in predicting GERD. Additionally, if both maneuvers are performed, the SUT maneuver complements the SLR in predicting pathologic GERD.

The basic concept underlying the SLR maneuver is the “common cavity” phenomenon, first described by Butterfield et al. in 1972, who observed increased IEP during elevated IAP in symptomatic patients with suspected EGJ impairment [20, 21]. The SUT maneuver creates an increase in IAP similar to the SLR maneuver, and consequently, a similar increase in IEP when the EGJ barrier is impaired. The underlying principle is that an increase in intra‐gastric pressure from increased IAP during either of these maneuvers would challenge and stress the EGJ. A competent EGJ barrier prevents backflow of gastric contents from the high‐pressure intra‐abdominal stomach to the low‐pressure intra‐thoracic esophagus [19]. When the distal esophagus lies in the abdomen, an increase of the intra‐abdominal pressure works as an “external reinforcement” of the EGJ to counteract similar increases in the intra‐luminal gastric pressure [28]. On the contrary, in patients with a disrupted EGJ, an IAP increase creates a virtual “common cavity” that promotes a retrograde movement of refluxate [20]. Our findings reveal that the SUT maneuver generated a greater IAP increase compared with SLR, and consequently was more frequently effective (85.5% vs. 77.3%). This suggests that the position change required by CCv4.0 could be leveraged as a built‐in provocative maneuver, potentially streamlining the HRM protocol while maintaining diagnostic accuracy. This may be particularly beneficial for older adults or patients with physical limitations, who may find SLR challenging due to reduced muscle strength or coordination. Future updates to the CCv4.0 protocol could consider incorporating SUT as a standardized provocative maneuver, pending further validation of reference values and standardization protocols.

The SUT demonstrated higher sensitivity (77.4% vs. 71.0%) but lower specificity (63.5% vs. 79.6%) compared to the SLR in predicting pathologic GERD. These findings could partly be related to the difficulty in standardization of the SUT maneuver, while the SLR has definitive steps in how the maneuver is performed. Another possible reason could be that both maneuvers have been evaluated using SLR cut‐off values, as the SUT reference values have not yet been validated. In any case, this trade‐off between sensitivity and specificity has important clinical implications. The higher sensitivity of the SUT suggests it may be particularly valuable as a screening tool, helping to identify patients who might benefit from further diagnostic evaluation. However, its lower specificity indicates that positive results should be interpreted with caution and may require confirmation through additional testing or alternative maneuvers.

When both maneuvers were integrated into the Milan Score, their predictive performance was comparable, with areas under the ROC curve of 0.825 for SUT and 0.854 for SLR. This suggests that the SUT could potentially replace the SLR in the Milan Score calculation without significantly compromising its diagnostic accuracy. Furthermore, in cases where both maneuvers were effective and concordant (47% of patients), the diagnostic accuracy improved substantially, with sensitivity and specificity reaching 88.2% and 80.0%, and an AUC for the Milan Score of 0.872. This observation suggests that using both maneuvers in combination, when feasible, might provide the most reliable assessment of EGJ barrier function.

The study has several strengths, including its multicenter design and the prospective data collection. The participation of centers across Europe, North America, and Asia enhances the generalizability of our findings. Additionally, the use of standardized protocols and definitions aligned with current international consensus (CCv4.0 and Lyon 2.0) strengthens the validity of our results.

Our study findings have to be tempered by a few limitations. First, the SUT maneuver's reliance on self‐initiated positional change introduces variability due to differences in muscle strength, balance, and coordination, which may be pronounced in older adults or patients with impaired functional status. Some patients might require assistance during the transition, which could affect the standardization of the maneuver. This variability could affect the degree of IAP generated, potentially introducing bias in EGJ function assessment. In our study, we did not assess functional status, which may further influence SUT performance. Moreover, while SUT could potentially reduce HRM procedure time and improve patient comfort, we did not measure maneuver duration or patient‐reported comfort as this study was focused on diagnostic performance. Future studies should quantify these outcomes to confirm the benefits of SUT in clinical practice and should focus on establishing standardized protocols for executing and measuring the SUT maneuver to ensure reproducibility across different centers and operators. In the study population, the rate of patients with GERD is fairly low (35.5%). Larger studies with a higher number of GERD patients are needed to confirm these results. Moreover, since the majority of patients were studied with the Manoview system (87.3%), the same study needs to be replicated with other HRM systems. Lastly, the potential inter‐operator variability in performing and interpreting SUT and SLR maneuvers across seven international centers may introduce another limitation, as no formal shared training or centralized analysis was performed.

In conclusion, the SUT maneuver could potentially simplify the HRM protocol by utilizing a position change that is already required, thereby reducing the total examination time and patient discomfort. This could be particularly beneficial in patients who have difficulty performing the SLR due to physical limitations. Furthermore, the complementary nature of the two maneuvers suggests that using both, when possible, might provide the most comprehensive assessment of EGJ barrier function.

## Author Contributions

All authors approved the final version of the manuscript.

## Conflicts of Interest

The authors declare no conflicts of interest.

## Supporting information


**Figure S1.** Demographic, endoscopic, HRM and pH‐impedance key differences between patients with effective and non‐effective SLR and SUT. Values are reported as a percentage of the total population (%). Statistically significant differences (*p*‐value < 0.05) are marked with *. EGJ, esophago‐gastric junction; GERD, gastro‐esophageal reflux disease; HRM, high‐resolution manometry; IEM, ineffective esophageal motility; SLR, straight leg raise; SUT, supine‐upright transition.

**Figure S2.** Relationship between age, BMI and the SUT and SLR maneuvers.

## Data Availability

Individual participant data will not be shared.
