# Supplementary figures and images for: Assessment of Esophagogastric Junction Barrier Function With the Supine‐Upright Transition of the Chicago Classification Protocol
Source: Neurogastroenterol Motil. 2025 May 26;37(12):e70088. doi: 10.1111/nmo.70088 (PMC12623285; doi:10.1111/nmo.70088)

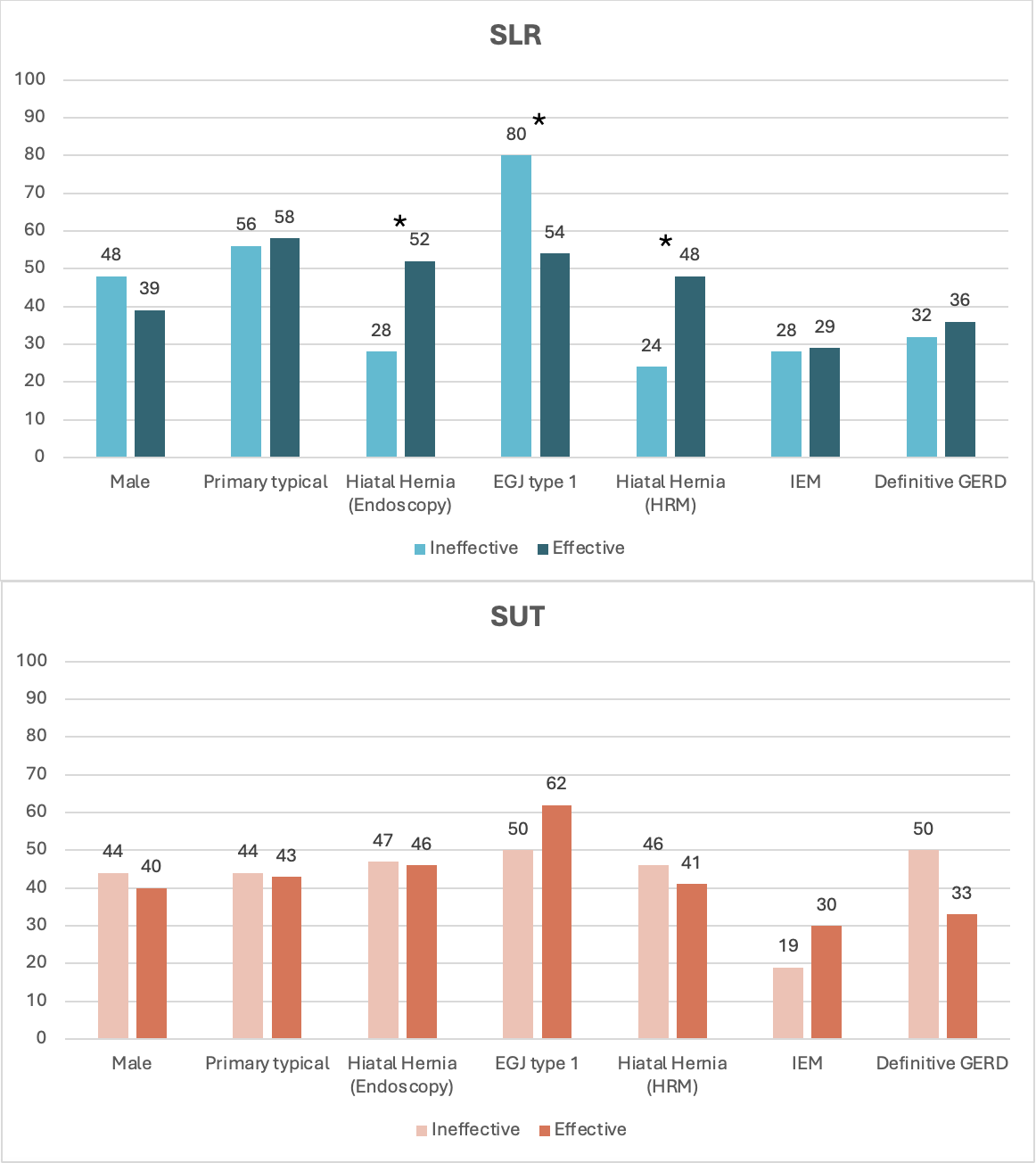

Supplement: Supplementary file 1 — Figure S1. Demographic, endoscopic, HRM and pH‐impedance key differences between patients with effective and non‐effective SLR and SUT. Values are reported as a percentage of the total population (%). Statistically significant differences (p‐value < 0.05) are marked with *. EGJ, esophago‐gastric junction; GERD, gastro‐esophageal reflux disease; HRM, high‐resolution manometry; IEM, ineffective esophageal motility; SLR, straight leg raise; SUT, supine‐upright transition. [file NMO-37-e70088-s002.tiff]

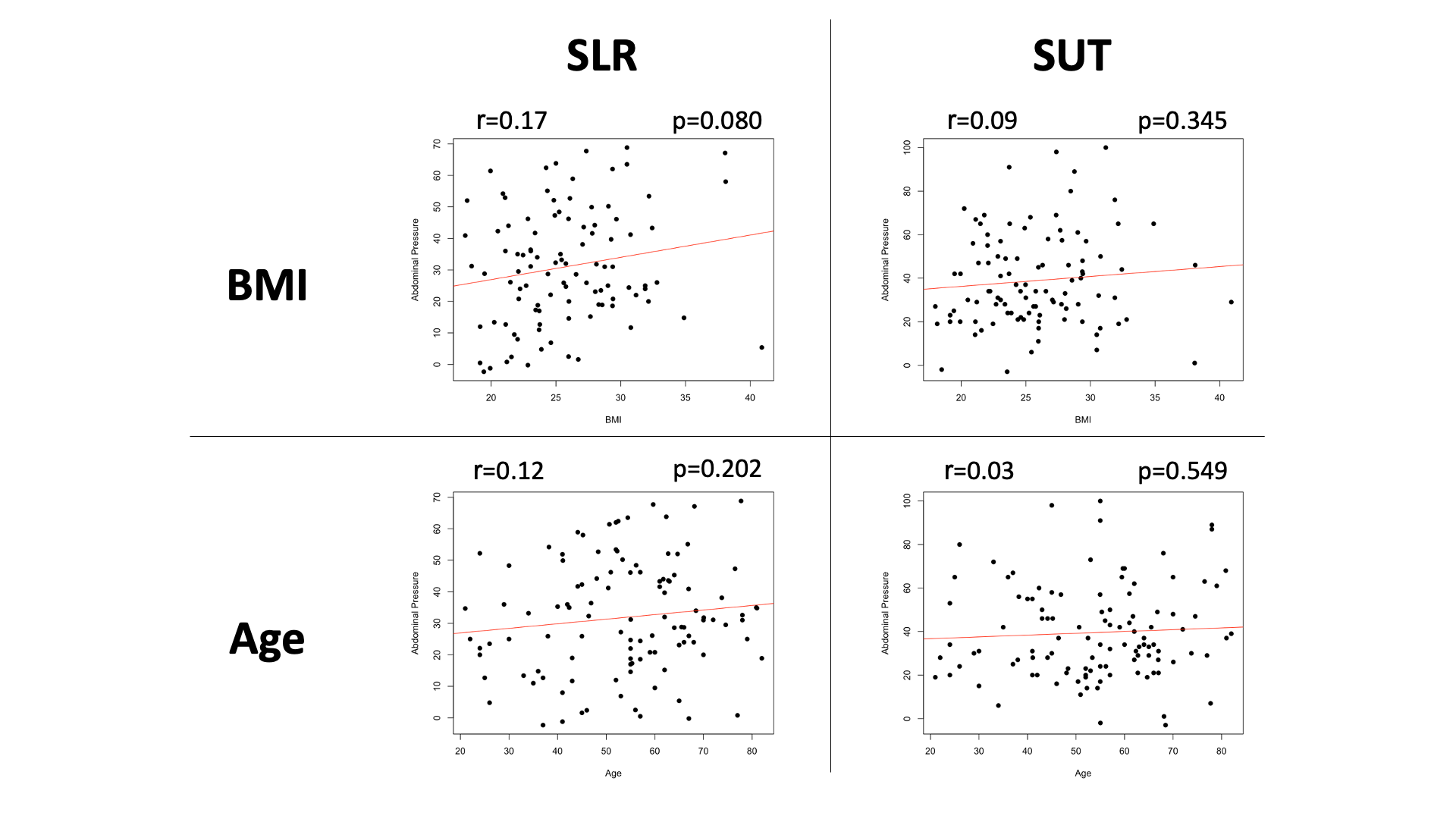

Supplement: Supplementary file 2 — Figure S2. Relationship between age, BMI and the SUT and SLR maneuvers. [file NMO-37-e70088-s001.tiff]
